# A Case of Euglycemic Diabetic Ketoacidosis (DKA), Influenza, and a Dipeptidyl Peptidase-4 (DPP-4) Inhibitor

**DOI:** 10.7759/cureus.39012

**Published:** 2023-05-14

**Authors:** Su Hyun Jeong, Merica Vorachitti, Fernando Fuentes

**Affiliations:** 1 Internal Medicine, Renown Health, Reno, USA; 2 Internal Medicine, University of Nevada Reno School of Medicine, Reno, USA; 3 Pulmonology, Renown Health, Reno, USA

**Keywords:** dpp4 inhibitor, sitagliptin, diabetes, endocrine, endocrinology, euglycemic dka

## Abstract

A subclass of diabetic ketoacidosis (DKA) is euglycemic DKA, characterized by the same traits of ketoacidosis and low bicarbonate levels. However, the condition differs from classic DKA because of its normoglycemic levels. Euglycemic DKA was once thought to be incredibly rare, but its incidence has been on the rise with the widespread use of sodium-glucose-cotransporter-2 (SGLT2) inhibitors and other newer anti-diabetic medications. The disorder is not fully understood and is often missed when presenting because of the non-elevated blood sugars. Common triggers for euglycemic DKA include infection, fasting, pregnancy, and medications such as SGLT2 inhibitors. This case report involves a patient with type 2 diabetes mellitus on sitagliptin that presented to the emergency department with shortness of breath, cough, nausea, vomiting, and abdominal pain and tested influenza positive with blood glucose levels of 209 mg/dl. He was started on IV fluids and subcutaneous insulin but developed worsening acidosis. The following day, he was transferred to the ICU for DKA protocol and diagnosed with euglycemic DKA.

## Introduction

Diabetic ketoacidosis (DKA) is a severe complication of diabetes mellitus that occurs from an absolute or relative insulin deficiency, which prevents the body from utilizing glucose. This causes the body to instead break down fat for energy. This breakdown of fat results in a subsequent rise in ketones and eventual metabolic acidosis. Per the American Diabetes Association, some diagnostic criteria include a plasma glucose concentration above 250 mg per dL, a pH level less than 7.30, and a bicarbonate level of 18 mEq per L or less [1]. However, there are rare incidences where a patient can experience DKA without elevated plasma glucose. This phenomenon is referred to as euglycemic DKA. The condition is not completely understood but is often associated with sodium-glucose-cotransporter-2 (SGLT-2) inhibitors, fasting, and pregnancy [2].

Dipeptidyl peptidase-4 (DPP-4) inhibitors are a class of glucose-lowering medications that work to prevent the inactivation of glucagon-like-peptide-1 (GLP-1) by inhibiting DPP-4 in peripheral plasma [3]. GLP-1 propagates glucose-dependent insulin secretion from pancreatic islet cells by inhibiting glucagon release. This medication is classically utilized for type 2 diabetics and typically packaged with metformin or a statin when prescribed. The current FDA-approved DPP-4 inhibitors include sitagliptin, saxagliptin, linagliptin, and alogliptin.

DPP-4 inhibitors have their drawbacks, including nausea, hypersensitivity reaction, headache, etc. Acute pancreatitis is a serious reported side effect with a 2017 study finding a 57% increased risk of acute pancreatitis with DPP-4 inhibitors compared to placebo [4]. DKA is a very uncommon side effect with a 2013 study finding a rate of 2.3 events per 1000 person-years using DPP-4 inhibitors [5]. For euglycemic DKA, the incidence is even rarer.

In this report, we present a case of a patient with diabetes mellitus on sitagliptin that developed euglycemic DKA after a recent influenza infection.

## Case presentation

Our patient is a 76-year-old male who presented with shortness of breath and a cough. One day prior to admission, he developed symptoms of nausea, vomiting, loose stools, hiccups, and mild abdominal pain for which he later presented to an urgent care center and was promptly told to go to the emergency department for further evaluation. He has a history of type 2 diabetes mellitus on sitagliptin 50 mg tab daily, metformin 1000 mg twice a day, and glargine 20 units at night with the potential for non-compliance at times, although he endorses that he takes all his pills.

On presentation, vital signs were as follows: blood pressure of 159/84 millimeters of mercury (mmHg), pulse of 95 beats per minute, respiratory rate of 18 breaths per min, temperature of 36.8 degrees Celsius, and saturation of peripheral oxygen at 98% on room air. He was found to have metabolic acidosis with an elevated anion gap of 21.0 and plasma bicarbonate of 18 mmol/L where he was initially treated with IV fluids and subcutaneous insulin on the medical floor. He then continued to develop nausea and vomiting which lead to concern for euglycemic DKA in the setting of the oral medications he was taking at home. He was transferred to the ICU but was ultimately not placed on an insulin drip because the point of glucose testing at the bedside was 160mg/dL. Although not a guideline therapy, there were concerns regarding the patient's normoglycemia. Additional labs showed an elevated beta-hydroxybutyric acid of 5.98 mmol/L, sodium 134 mmol/L, potassium 4.1 mmol/L, chloride 96 mmol/L, bicarbonate 14 mmol/L, glucose 154 mg/dL, and an anion gap of 24. Further labs are shown below in Table 1.

**Table 1:** Hospital course labs Basic labs starting from the day of admission to the day of discharge. Beta-hydroxybutyrate was not collected until there was elevated clinical suspicion of euglycemic DKA

	Day 1: Admission	Day 2: ICU transfer	Day 6: Discharge	Reference ranges
Sodium	133	134	136	135-145 mmol/L
Potassium	4.4	4.1	3.9	3.6-5.5 mmol/L
Chloride	94	96	102	96-112 mmol/L
Co2	18	14	22	20-33 mmol/L
Anion gap	21	24	12	
Glucose	209	154	169	65-99 mmol/L
Beta hydroxybutyrate		5.98		
Lipase		87		7-58 U/L

Diagnostic work-up with chest X-ray showed no evidence of acute process. He was started on a bicarbonate drip until acidosis resolved and the anion gap closed. There were no further signs of infection other than his positive Influenza A for which he was on oseltamivir with normal lactic acid. There were no clinical concerns for pancreatitis as his initial abdominal pain was related to the hiccups which were resolved with baclofen, and CT of the abdomen showed no signs of inflammation although lipase was mildly elevated at 87 U/L. During his hospitalization, beta-hydroxybutyrate decreased to 1.29 mmol/L, and the anion gap improved to 12 with IV fluids, subcutaneous insulin, and bicarbonate drip without the need for an insulin drip.

## Discussion

Euglycemic DKA is uncommon, with a prevalence of 2.6-3.2% of DKA admissions being normoglycemic [6]. The literature is sparse when it comes to the relationship between euglycemic DKA and DPP-4 inhibitors, but reports indicate the development of acute pancreatitis prior to presentation. One case involving a 38-year-old patient with type 1 diabetes mellitus with end-stage renal disease presented with euglycemic DKA after developing acute pancreatitis from his sitagliptin [7]. A second case involving a 58-year-old patient with type 2 diabetes mellitus on a DPP-4 inhibitor developed euglycemic DKA from pancreatitis [8]. Both these cases hypothesize that the sitagliptin had a counter-regulatory effect on the physiologic response that causes hyperglycemia in classic DKA. The authors presumed the DPP-4 inhibitor controlled glucose levels by prolonging the effects of GLP-1, which in turn upregulated incretins and decreased glucagon.

The presentation of our case differs from both these cases as our patient did not meet the diagnostic criteria for acute pancreatitis. Our patient had a mildly elevated lipase level of 87, abdominal pain, and no acute abnormalities on CT. The evidence is mixed, but the mechanism between DPP-4 inhibitors and pancreatitis is believed to be from an increased state of chronic inflammation in the pancreas from the prolonged effects of GLP-1 agonism. This in turn causes ductal hyperplasia with outflow obstruction and increased activation of pancreatic enzymes, damaging beta cells [9]. The subsequent harm to these pancreatic beta cells blunts the secretion of insulin [9]. In more severe cases, this would naturally cause a rise in glucose levels, but we assume for our patient his pancreatic beta cell function remained intact enough to prevent hyperglycemia. Nevertheless, this patient’s ketoacidosis resolved after the initiation of insulin, which supports the hypothesis of pancreatic beta-cell injury.

Additionally, unlike the other reports, our patient tested positive for influenza. Viral infections are often associated with DKA with studies showing increased incidence of DKA during pandemics [10]. The pro-inflammatory state caused by influenza has been shown to both elevate blood sugars by impairing insulin sensitivity and elicit more severe symptoms in patients with diabetes mellitus. This in turn can indirectly cause ketoacidosis from poor oral intake due to viral symptoms of vomiting, nausea, abdominal pain, etc. Sitagliptin’s effect on insulin certainly would combat influenza’s effect on hyperglycemia. However, evidence suggests the medication has an additional anti-inflammatory element by suppressing CD26, a mediator of inflammatory signaling [11]. The suppressed inflammatory response would reduce the severity of our patient’s infection, decreasing his hyperglycemia.

## Conclusions

Euglycemic DKA is an often-missed clinical diagnosis with serious consequences if not immediately identified and treated. Out of the anti-diabetic drug classes, the condition is most associated with SGLT-2 inhibitors. However, more cases are being reported involving other classes of anti-diabetic drugs including DPP-4 inhibitors. When treating diabetic patients with ketoacidosis in the absence of hyperglycemia, providers should be careful not to exclude DPP-4 inhibitors as a potential cause and discontinue them.
